# Primary graft dysfunction after heart transplantation: a thorn amongst the roses

**DOI:** 10.1007/s10741-019-09794-1

**Published:** 2019-04-25

**Authors:** Sanjeet Singh Avtaar Singh, Jonathan R. Dalzell, Colin Berry, Nawwar Al-Attar

**Affiliations:** 1 Department of Cardiothoracic Surgery, Golden Jubilee National Hospital, Glasgow, Scotland; 2 Scottish National Advanced Heart Failure Service, Golden Jubilee National Hospital, Glasgow, Scotland; 3 Institute of Cardiovascular & Medical Sciences, University of Glasgow, Glasgow, Scotland

**Keywords:** Primary graft dysfunction, Pathophysiology, Treatment, Management, Prevention

## Abstract

Primary graft dysfunction (PGD) remains the leading cause of early mortality post-heart transplantation. Despite improvements in mechanical circulatory support and critical care measures, the rate of PGD remains significant. A recent consensus statement by the International Society of Heart and Lung Transplantation (ISHLT) has formulated a definition for PGD. Five years on, we look at current concepts and future directions of PGD in the current era of transplantation.

## Primary graft dysfunction

Heart transplantation remains the closest resemblance to a ‘cure’ for end-stage heart failure. Worldwide, more than 4000 adults undergo heart transplantation annually [1]. Whilst survival after cardiac transplantation has improved over the past four decades, primary graft dysfunction (PGD) is the leading cause of 30-day mortality post-transplant. The incidence of PGD is yet to be accurately delineated given the lack of an international consensus regarding its definition. Reports to date vary widely with respect to diagnostic criteria and definitions, therefore making incidence and outcomes difficult to compare between centres and regions. Prior to 2014, the International Society of Heart and Lung Transplantation (ISHLT) registry noted a 30-day of 10% in all heart transplants done since 1982 [2]. The 90-day mortality was 14%. Almost 70% of these deaths were coded as ‘graft failure’ or ‘multi-organ failure’ for which a large majority would likely constitute contemporary PGD. Here, we provide a comprehensive overview of PGD in heart transplantation.

### Definition

PGD presents as severe ventricular dysfunction of the donor graft which fails to meet the circulatory requirements of the recipient in the immediate post-transplant period. It may manifest as either single or biventricular dysfunction with low cardiac output and hypotension despite adequate filling pressures [3–5]. In 2014, Kobashigawa et al. provided a consensus definition and grading system based on the modified Delphi method [6]. This agreement upon a uniform definition allowed subsequent studies to outline its true incidence and further explore and identify the potential multifactorial aetiologies underpinning PGD.

PGD is defined as being a separate entity from secondary graft dysfunction, which is when a discernible cause for allograft dysfunction is identified. Such causes can include hyperacute rejection, graft dysfunction secondary to pulmonary hypertension or a recognised intraoperative complication. The diagnosis of PGD is made within 24 h post-transplantation and is separated into PGD-LV, for PGD affecting the left ventricle or biventricular failure; and PGD-RV for isolated right ventricular involvement [6]. A severity scale applies to PGD-LV. For the mild and moderate categories, it relies on the requirement of inotropic support with a composite score as described by Wernovsky et al. (< 10 = mild, ≥ 10 = moderate) alongside either using echocardiography to identify ventricular dysfunction or right heart catheterisation to demonstrate haemodynamic compromise [7]. For the echocardiography criteria, the left ventricular ejection fraction < 40% is considered diagnostic for PGD (in the absence of secondary causes). With regard to haemodynamic parameters, high filling pressures i.e. a right atrial pressure (RAP) > 15 mmHg, and pulmonary capillary wedge pressure (PCWP) > 20 mmHg indicate PGD if occurring in the context of a low cardiac index (CI) (< 2.0 L/min/m^2^) lasting at least 1 h. The requirement of an intra-aortic balloon pump signifies moderate PGD-LV, whereas requirement of extracorporeal short-term mechanical circulatory support in the form of extracorporeal membrane oxygenation (ECMO) or ventricular assist devices (VADs) in any form (percutaneous/surgical) is diagnostic of severe PGD-LV [6].

PGD-RV does not have a severity scale and is diagnosed based on the requirement of a right-sided short-term VAD (RVAD) or right heart catheter measured haemodynamics in keeping with isolated right-sided dysfunction (RAP > 15 mmHg, PCWP < 15 mmHg, CI < 2.0 L/min/m^2^, TPG < 15 mmHg and/or pulmonary artery systolic pressure < 50 mmHg) [6].

Previous studies had used the need for mechanical circulatory support as a criterion for diagnosis of primary graft dysfunction; however, the timing of initiation of therapy and endpoints differs from these studies, leading to significant variation in incidence reporting (2.3–32.4%) [4, 8–11]. With an increasing trend towards utilisation of marginal donor organ due to increasing waiting list pressures, a resultant reduction in threshold for initiation of ECMO support in certain patients to support the graft in the initial phase of reperfusion (first 24 h) may result in an over-estimation of the incidence of PGD [12]. This is especially true in high-risk recipients with a significant inflammatory milieu as a result of receiving a combination of in-hospital inotropic or short- or long-term mechanical circulatory support pre-transplant.

### Pathophysiology of PGD

The donor heart is exposed to a multitude of physiological insults at four specific time points: brainstem death, hypothermic ischaemia, warm ischaemia and ischaemia-reperfusion injury.

#### Brainstem death

The first insult occurs during declaration of brainstem death in donation after brainstem death (DBD). Raised intracranial pressure (ICP) invariably is the final pathway in DBD donors in which case the aetiology of death is usually a result of an intracerebral haemorrhage, hypoxia leading to oedema, inflammation or a space occupying lesion in the cranium. The cerebral perfusion pressure is usually maintained by homeostatic mechanisms which result in an increased arterial pressure. Due to the limited plasticity of the brain within the confines of the cranium, brain herniation ensues resulting in pontine ischaemia [13]. This causes a surge in the adrenergic response, resulting in pulmonary and systemic hypertension which increases the afterload in both ventricles causing myocardial ischaemia. In some patients, stimulation of baroreceptors results in the classic Cushing’s response characterised by hypertension and bradycardia [14, 15].

Vasomotor tone is reduced to the loss of spinal cord sympathetic activity resulting in unopposed vasodilatation, further reducing preload and indirectly afterload, affecting coronary blood flow and reducing myocardial perfusion. As a response, there is an intense release of myocardial noradrenaline immediately after brain death that results in mitochondrial and cytosolic calcium overload to increase contractility to counteract this unopposed vasodilatation [15]. Pituitary ischaemia may also occur during brain herniation resulting in significant endocrine derangement [16].

These patients are also prone to metabolic disturbances due to acidosis, hypomagnesaemia or hypokalemia secondary to mannitol initiation to reduce ICP, catecholamine administration, all of which contribute to increased myocardial oxygen demand or reduced myocardial perfusion [16]. This initial surge may also cause catecholamine depletion leading to a vicious circle of impaired myocardial oxygenation with increasing myocardial oxygen demand [13].

Early donor management may reduce the deleterious effects of the abovementioned phenomenon. Early administration of vasopressors is aimed at reducing the unopposed vasodilatation [17]. Methylprednisolone administration has been shown to reduce lung injury independently from its anti-inflammatory activity in animal studies as well as reduce extravascular lung water and reduce PCWP in DBD donors [18, 19]. It is also used in conjunction with insulin to ensure normoglycemia in the donor [20]. The role of thyroid hormone replacement is uncertain during donor management with current consensus suggesting triiodothyroxine (T_3_) administration only in depleted donors [16, 19, 21, 22].

#### Hypothermic ischaemia

Despite advancements in normothermic ex vivo perfusion devices, cold storage remains the mainstay of transportation after organ retrieval. Organ retrieval often has two distinct phases, a warm phase and a cold phase. The warm phase involves dissection of the heart and exposure of the innominate artery and the two cavae with variations in the degree of dissection depending on whether the lungs are to be retrieved as well. The retrieval surgeon also palpates the coronary arteries for potential disease. The aorta is prepared for administration of cold cardioplegia for commencement of the cold phase.

Depending on the size of the patient and type of cardioplegic solution administered, the volume of administration may differ. The retrieval surgeon then performs a left atriotomy by lifting the heart. Once the aortic cross-clamp is applied, cold cardioplegia is infused via the aortic root at approximately 4 °C. The retrieval process is completed with the heart placed in a cold storage container. Cold storage induces hypothermic arrest of metabolism and maintains viability during this reduced metabolic state, therefore abating cellular swelling and minimalizing reperfusion injury [23]. At these temperatures, and with limited oxygenation, the heart switches from aerobic to anaerobic metabolism. This can have deleterious effects on the stored organs as ATP is slowly depleted; however, it should be noted that in the hypothermic state (0–4 °C), there is a 12-fold decrease in metabolic rate [24]. The overall goal is to reduce the accumulation of mitochondrial byproducts of metabolism such as oxygen free radicals. Cold storage is also based on the assumption that there is no variation in temperature and the heart is uniformly cooled. In elderly patients whereby, pathologic LV hypertrophy may be noted especially in those with a clinical history of hypertension this may not be possible, possibly explaining why these hearts are more susceptible to ischaemic injury [4, 25]. The length at which the hearts are kept in cold storage may also play a part in the formation of these free radicals. Cellular swelling and lactic acidosis occurs in prolonged cold storage, causing a rise in intracellular H^+^ ions [26]. The Na^+^/H^+^ exchanger is activated resulting in an increase in intracellular Na^+^ which activates the Na^+^/Ca^2+^ exchanger [27, 28]. The final pathway is the accumulation of cytosolic Ca^2+^. This will play a major role in the pathogenesis of ischaemic-reperfusion injury.

#### Warm ischaemia

On arrival at the recipient centre, the heart is removed from cold storage and inspected. The period in which the heart is removed from cold storage or from ex vivo normothermic perfusion to the final release of the recipient aortic cross-clamp is termed warm ischaemic time. The heart is exposed to warmer temperatures which slowly increases its metabolic rate resulting in increased formation of oxygen free radicals. In a study by Marasco et al., warm ischaemic time and increasing donor age were independent predictors of early survival, suggesting an acceleration of the deleterious effects described during the cold ischaemic phase [29]. Similar findings were noted by Banner et al., despite a broader definition of warm ischaemic time termed surgical implant time (i.e. arrival of heart to theatre to release of recipient aortic cross-clamp).

#### Ischaemia-reperfusion injury

Ischaemia-reperfusion injury (IRI) is defined as cardiomyocyte damage secondary to myocardial restoration of blood flow [30]. On release of the recipient cross-clamp, the donor heart is re-perfused which leads to further calcium overload. This alongside the release of oxygen free radicals activates the formation of mitochondrial permeability transition pores (MPTP) which are non-specific, thus allow free movement of apoptotic factors across the cell membrane [31]. This causes a cascade of myocardial damage, causing loss of cardiomyocyte function and viability; all of which are most pronounced immediately after reperfusion [32]. Post-conditioning may play a role in attenuating the effects of IRI by inhibiting MPTP formation. Cyclosporine A, a known MPTP desensitiser, has been shown to induce appreciable protection in acute myocardial infarction [33]. However, a recent meta-analysis failed to show any benefit of cyclosporine A post-infarction [34]. Other forms of preconditioning have also been suggested in an attempt to inhibit MPTP formation have shown some promise in animal studies [35–37]. However, larger clinical trials in surgical revascularisation studies have revealed mixed results with some studies showing clinical improvements [38] and others showing minor biochemical improvements with no clinical benefit [39–43]. The proponents of ischaemic preconditioning have suggested that the duration of ischaemia in these studies probably limits the effect of preconditioning with prolonged ischaemia showing greater benefit of preconditioning [44]. To date, no studies have been performed in human heart transplants, with animal models of orthotopic heart transplants showing promising results potentially highlighting its benefits in prolonged ischaemia [45].

Animal studies do however highlight attenuation of IRI using preconditioning in diabetics [46–48], females [49, 50], LV hypertrophied hearts [51], obesity [48], hypertensives [52] and in the elderly [50, 52, 53]; all of which have been noted to be risk factors for PGD.

Another mechanism of IRI is hypercontracture-mediated sarcolemmal rupture (HMSR). During the ischaemic phase, the low cytosolic ATP concentrations are quickly exhausted resulting in myofibrillar shortening that remains fixed as all cross-bridges between actin and myosin remain in an attached state [54]. This causes a moderate contracture with little structural damage but leads to cytoskeletal defects and increasing the cardiomyocyte fragility to mechanical damage which is reversible with early reperfusion [55].

Reperfusion-induced hypercontracture occurs after prolonged ischaemia. There is greater myofibrillar shortening and cytoskeletal damage when compared to the ischaemic phase alone. It causes a marked rise in end-diastolic pressure with increased ventricular wall stiffness. It is shown to be due to Ca^2+^ overload which develops during ischaemia and is rapidly re-energised, as it would on release of the cross-clamp [56]. In cellular studies, re-perfused infarcts consist almost exclusively of contraction band necrosis [56]. Sarcolemmal rupture occurs due to the degradation of structural proteins like α-fodrin [57] and ankyrin [58], impairing the Na^+^/Ca^2+^ exchanger pumps. Sarcolemmal rupture also results in increasing Na^+^ influx into cardiomyocytes via gap junctions and may propagate to adjacent cells [59].

#### Donation after circulatory death

Donation after circulatory death (DCD) provides a different set of insults to the donor heart. The heart is placed in a hypoxemic and hypercarbic environment following withdrawal of life support [60]. This causes pulmonary vasculature constriction, with increased pulmonary vascular resistance imposed on the right heart [61]. Although the catecholamine surge of brainstem death is not present, the hypoxemic environment induces an adrenergic response. This quickly depletes the myocardial energy stores—leading to circulatory arrest. A mandatory standoff period ensues as part of the guidelines for declaration of circulatory death [62]. The heart is exposed to a period of warm ischaemic time that is present until the institution of either normothermic regional perfusion (NRP) or ex vivo normothermic perfusion using the Organ Care System device (TransMedics Organ Care System (OCS), Andover, MA, USA) [63].

This prolonged ischaemic period causes intracellular acidosis which causes activation of the Na^+^/H^+^ exchanger in a pathway similar to the warm ischaemic period described above, with a net result of an accumulation of intracellular Ca^2+^ and follows the ischaemic-reperfusion pathway on reperfusion.

#### Recipient factors

Some recipient factors may also predispose a patient to PGD. Recipients, especially patients who have been on inotropic support and mechanical devices, have activation of their systemic inflammatory response, which causes intense vasodilation and therefore lowering of the systemic vascular resistance. The net result is a hypotensive state despite adequate filling and a high cardiac output that is refractory to vasopressor support [64]. The exact pathogenesis of this is poorly understood to date, some mechanisms have been postulated. Vasodilation is believed to be caused by the unopposed activation of vascular smooth muscle adenosine triphosphate-sensitive potassium channels (K_ATP_ channels). Endogenous nitric oxide, a potent vasodilator, is also implicated in the pathogenesis alongside vasopressin deficiency [65].

Prolonged ischaemic time has been shown in previous studies to correlate with complications occurring after cardiopulmonary bypass [64]. This association is thought to be due to the summative effect of the exposure of blood to synthetic surfaces of the cardiopulmonary bypass circuit which potentiates a cascading inflammatory response. This in turn could explain the increased incidence in recipients on advanced mechanical circulatory support (ECMO or Short-Term VAD). The ischaemic time may also be prolonged in some patients whereby careful dissection is necessitated following a previous sternotomy, highlighting the importance of communication with the retrieval team to potentially delay the retrieval procedure to minimise ischaemic time [66].

Another pathogenic mechanism that has been suggested in the past is recipient pulmonary hypertension. The donor heart in its ischaemic state is exposed to a relatively high pulmonary vascular resistance, giving rise to right-sided heart failure. Due to the poor adaptability of the right ventricle to a sudden change in vascular resistance, biventricular dysfunction ensues due to a reduced left-sided preload, thereby reducing coronary perfusion and end organ perfusion in the systemic circulation [67]. This however is currently termed secondary graft dysfunction and is beyond the scope of this review.

### Risk factors for PGD

There are several risk factors that have been suggested that may contribute to PGD. These include donor factors, recipient factors and procedural factors.

#### Donor factors

##### Donor age

Avtaar Singh et al. (2018) highlighted donor age to be a significant risk factor on multivariate analysis. This may have been due to the poorer tolerance for long ischaemic times in hearts from older donors. They highlighted an odds risk of 20% for each decade increment in donor age. A similar finding was noted by Russo and colleagues [68]. They noted that in younger donors, there was not significant correlation between ischaemic time and survival. However, as the donor age increased, there was a statistically significant correlation between ischaemic times and survival in donor ages > 20. Other studies have also shown a direct correlation between donor age and low cardiac output states post-operatively, which were likely PGD, however were not characterised as such due to the lack of a definition at the time of publication [69–72].

##### Gender mismatch

Female-donor to male-recipient gender mismatch is a risk factor for PGD. The exact mechanism is poorly understood as this persisted despite organ size matching. Male-donor to female-recipients did not result in an increased PGD rate. Gender-mismatched organs also had a poorer survival at up to 5 years. In liver transplantation, recipients had poorer graft survival in female-donor/male-recipient cohorts compared with other donor-recipient gender groups despite adjustment for donor risk factors and recipient variables [73]. The role of H-Y minor histocompatibility antigens has been implicated. Several haemotopoetic stem cell studies have shown an increased relapse rate in patients undergoing stem cell transplants in female-donor/male-recipient cohorts [74, 75].

##### Cause of death in the donor

Intracranial haemorrhage is the most common cause of death for DBD donors in the UK. One study showed increased PGD rates in organs retrieved from donors with intracranial haemorrhage compared to donors with traumatic cause of death [76]. The exact mechanism is not clear, although it is thought to be due to a prolonged exposure to the catecholamine surge [76]. Animal studies have shown a direct correlation between increased intracranial pressure and worsening myocardial function [15, 77]. Avtaar Singh et al. highlighted intracranial haemorrhage as a risk factor on the unadjusted analysis although it was not present during multivariate analysis. Another study showed an association with early mortality with a lower survival to discharge rate in patients transplanted with hearts from donors with atraumatic intracranial haemorrhage [78].

##### Donor left ventricular hypertrophy

Donors with hypertension often have a degree of left ventricular hypertrophy (LVH). This is more apparent across Europe where the average age of the donor is older compared to North America [1]. The use of donors with LVH has resulted in mixed results with some centres having favourable results [25, 79–82] but others reporting an increased incidence of early graft failure or poorer survival [83–85]. The reason for the varying results is likely due to the association of LVH to age, which is an independent predictor of PGD. Hypertrophied hearts with associated hypertension are more susceptible to ischaemic injury [86]. Therefore, the ischaemic time for donors with hypertrophied hearts should be kept to a minimum. The Harefield team reported their experience in using marginal donors with mild LVH with normothermic ex vivo perfusion [87]. They showed promising short-term outcomes when utilising marginal hearts with continuous normothermic perfusion using the OCS device. The ISHLT guidelines also cites level of evidence C for a class IIa recommendation stating that “it would seem appropriate to use hearts from donors with LVH and LV wall thickness <14 mm provided that it is not associated with ECG findings of LVH” [88].

##### Donor inotropic requirement

Inotropic support is sometimes needed during the donor organ retrieval process due to the depletion of catecholamine and/or a systemic inflammatory phenomenon as described above. Noradrenaline use can cause left ventricular dysfunction in the absence of brain death [89]. It also has a dose-dependent detrimental effect on the right ventricle in DBD donors [90–92]. The use of vasopressin and terlipressin is currently recommended as first-line treatment to reduce the noradrenaline requirement [93]. It treats two conditions which occur commonly in DBD donors, neurogenic diabetes insipidus and reduction in systemic vascular resistance [94]. Venkateswaran et al. showed that vasopressin use could result in the elimination or reduction of noradrenaline use in more than 50% of donors [95].

#### Recipient factors

##### Pre-operative mechanical circulatory support

Pre-operative recipient ECMO/VAD usage has also been linked to PGD post-operatively [4, 90, 96, 97]. Several factors have been suggested as potential causes which have been discussed under the pathophysiology section above.

A recent study by Truby and colleagues highlighted the role of continuous flow LVAD (CF-LVAD) in recipients as a risk factor for severe PGD [98]. This is of particular interest as CF-LVADs have contributed significantly to the growth and success of mechanical circulatory support for advanced heart failure for BTT and as destination therapy. CF-LVADs also account for more 95% of VAD implants currently [99]. Forty-five out of 56 (80.4%) patients with severe PGD were bridged to transplant (BTT) using CF-LVADs in Truby’s study [98]. It should be noted however there were significant other contributary factors as the severe PGD patients were older, had a higher percentage of previous amiodarone exposure, had higher creatinine levels, spent longer time on the waiting list and had a higher CVP/PCWP ratio which is possibly indicative of subclinical right ventricular dysfunction.

#### Recipient diabetes mellitus

The role of recipient diabetes as a predictor of PGD is evident in multiple studies [92]. The results of Avtaar Singh et al. however show that the 95% confidence interval (0.9993–4.1720) crosses the boundary of no effect for diabetes. Segovia’s study was unable to account for recipient diabetes as a predictor of PGD when using robust statistical analysis (overfitting of regression models [100]. However, recipient diabetes seems to be a predictor of graft loss within and beyond the first-year post-transplant [101]. This may be due to direct glucose-mediated endothelial damage, oxidative stress from superoxide overproduction and production of advanced glycation end-products, which may result in changes in endothelial permeability, excessive vascular protein deposition and altered blood flow. Taghavi et al. revealed diabetic recipients were older and would more likely receive organs from diabetic donors who were who themselves were more often females and older [102].

##### Recipient age

Several studies have associated advancing recipient age with PGD and mortality [90, 92]. A risk prediction model assessing in-hospital mortality for post-heart transplant patients included recipient age but utilised it as a categorical variable. The c-statistic of less than 0.7 however suggested poor performance [103]. ISHLT registry data also highlighted advancing recipient age with having slightly higher long-term mortality. This may probably reflect the higher incidence of comorbidities such as hypertension and diabetes in this cohort of patients as well [104]. Some studies have attributed this to the increased fatal infection rate in the elderly [105, 106].

##### Recipient re-sternotomy

A recipient re-sternotomy indicates previous surgery, most commonly for durable ventricular assist device implantation, previous congenital cardiac surgery or previous coronary bypass or valvular surgery [107]. The dissection process in recipient who has undergone a previous sternotomy often is more complex and hazardous with the potential for significant injury [108]. Recipient re-sternotomy has been linked to an increased risk of severe PGD in a recent study. A similar study showed that patients with prior sternotomies had an almost threefold increase for PGD risk [109]. The increased technical difficulty of the surgery may result in longer ischaemic times and is associated with higher rates of blood transfusion and subsequent need for reoperation for bleeding [109–111]. Patients with prior sternotomies were also more likely be older [109] and have spent longer time on the waiting list [109], possibly indicating durable VAD implantation [110] in these patients as a bridge to candidacy/transplantation.

##### Recipient pre-operative amiodarone therapy

Amiodarone is a class III antiarrhythmic used for a variety of arrhythmias including both ventricular, supraventricular and atrial tachyarrhythmias. Its mechanism of action is primarily by prolonging the refractory period myocardium. Arrhythmias are highly prevalent in advanced heart failure patients [112]. The evidence regarding PGD and the use of amiodarone in recipients appears to be conflicting. Studies have shown that patients who had received amiodarone before transplantation had significantly lower heart rates post-transplantation [113, 114], required atrial pacing for a longer time after transplantation [113] but had no increased inotropic requirements [113] and no increased mortality post-operatively [113–115]. Other studies have indicated a dose-dependent [116] or duration-dependent [117] link between amiodarone use and PGD post-transplant.

#### Procedural factors

##### Ischaemic time

In DBD donations, the ischaemic time consists of the placement of the donor aortic cross-clamp till the release of the recipient aortic cross-clamp [118]. For DCD donations, the ischaemic time is possibly underestimated with a period of functional warm ischaemia (starting when systolic blood pressure is less than 50 mmHg) till the heart is re-perfused on an ex vivo perfusion device (OCS) [63]. A second period of ischaemia ensues from removal of the heart from the OCS till release of the aortic cross-clamp from the recipient.

Cold ischaemic time consists of the time spent in cold storage and warm ischaemia ensues once the heart is removed from cold storage or the OCS rig till release of the aortic cross-clamp. Prolonged ischaemic time is associated with an increased rate of PGD [4, 29, 92, 119]. Banner et al. noted that longer ischaemia time was a risk factor for 30-day mortality after heart transplantation. They also noted that the surgical implant time (i.e. warm ischaemic time) was an independent risk factor for 30-day mortality [120]. Avtaar Singh et al. noted that warm ischaemic time/implant time to be a significant independent predictor of ISHLT defined PGD. The direct ischaemic insult of warm ischaemia is explained in the pathophysiology above. Marasco et al. also noted found that poorer survival with a warm ischaemia time (WIT) of > 80 min [29]. Ischaemic time is also closely related to donor age. Older donor organs are more susceptible to ischaemic injury compared to younger donor organs [68, 121].

##### Cardiopulmonary bypass time

Prolonged cardiopulmonary bypass (CPB) duration independently predicts post-operative morbidity and mortality after general cardiac surgery [122]. Kirklin and colleagues alluded to a systemic inflammatory response following CPB [123]. The mechanism of injury from CPB and ischaemic-reperfusion of the myocardium is similar, both producing a hyperdynamic circulatory state due to a low systemic vascular resistance, platelet and coagulation factor dysfunction, inflammatory pathway activation triggered by leucocytes and endothelial cells and finally cytokine release and formation of oxygen free radicals [124]. It is also linked to an increased blood product requirement which is associated with both infection and ischaemic post-operative morbidity, increased hospital stay, increased early and late mortality and increased hospital costs [125–127]. Therefore, prolonged CPB time may contribute to the worsening of the ischaemic injury caused by PGD.

##### Size mismatch

The size of the donor graft has many implications on the recipient. It is a powerful predictor of survival recipients receiving undersized grafts having an increased 1-year mortality, and a 36% increased mortality within the first 30 days [128]. Smits showed inferior survival in donors who were undersized by more than 20% using data from the Eurotransplant database [129]. This study however also showed an inferior survival amongst gender-mismatched donors, which were more likely to be undersized in female donors to male recipients. Patel et al. refuted the findings in an analysis of the UNOS database, suggesting increased pulmonary resistance as a confounder [130]. Another analysis of the UNOS database several years later concluded that donor/recipient BMI ratio < 0.75 was associated with increase in post-transplant mortality in univariate analysis but not in multivariate analysis, highlighting gender mismatch as a confounder [131]. Other multi-institutional studies have also highlighted donor-recipient weight difference to be significant, but only in older grafts which were gender mismatched [132]. Jayarajan and colleagues noted that donor weight mismatching by up to 40% had no bearing on outcome in non-gender mismatched patients [133]. The significance of size mismatching is hard to elucidate from studies but smaller single-centre data suggests poorer outcomes such as an increased length of stay [134] and increased LV hypertrophy [135]. The direct link between PGD and size mismatch however remains elusive.

### Investigations and biomarkers

The current definition for PGD is based on the treatment options utilised. Several biomarkers have been suggested as potential predictors of PGD, although to date none are used in routine care.

#### Inflammatory markers

The pro-inflammatory state accompanying PGD supports the use of inflammatory markers as potential predictive biomarkers. Tumour necrosis factor-α (TNF-α), interleukin-6 (IL-6), neutrophils and procalcitonin (PCT) have all been suggested as potential biomarkers.

##### TNF-α

Tumour necrosis factors are produced by lymphocytes and macrophages that cause cell lysis [136]. TNF-α has been implicated in the pathogenesis of numerous inflammatory conditions including arthritis [137], asthma, chronic obstructive pulmonary disease (COPD), acute respiratory distress syndrome (ARDS) [138], myocarditis [139] and congestive heart failure [140].

Birks and colleagues noted an increased expression of TNF-α in unused donor hearts due to poor function and compared them with donors with good ventricular function (used donors) and patients with advanced heart failure (HF) [141]. In lung transplantation, a recent study highlighted the role of intravascular donor monocytes in the pathophysiology of IRI and PGD in lungs [142]. Another group showed reduction in PGD rates post-lung transplantation after leukocyte depletion [143]. Venkateswaran et al. highlighted poorer biventricular function in donors with elevated levels of TNF-α using serum immunoassays [144].

A Brazilian group showed recipients receiving grafts from donors expressing lower concentrations of plasma soluble tumour necrosis factor receptors 2 (sTNFR2) and IL-6, required more inotropic support post-transplantation [145].

##### IL-6

Interleukin 6 (IL-6) is produced in response to infections and tissue injuries by stimulation of acute phase responses. It bridges the adaptive and innate immune responses and plays a major role in autoimmune conditions [146, 147]. Birks et al. noted IL-6 mRNA expression was 2.4-fold higher in the unused donor hearts than in those used for transplantation, with levels almost five times higher than in the potential recipients with advanced heart failure [141]. The used donor hearts had an almost twofold increase of IL-6 mRNA levels compared to the recipients. These findings were also noted by Plenz and colleagues, who noted a significant rise in IL-6 and IL-6 receptors in DBD donors, comparable to patients with advanced heart failure in comparison with a control group not compromised by the sequelae of brain death. This may explain the close association of elevated IL-6 serum levels and acute allograft dysfunction in the early post-operative period [148].

IL-6 was also shown to be associated with PGD post-lung transplantation. The serum and bronchioalveolar lavage concentrations of IL-6 were higher in transplant recipients with PGD than those without [149].

Animal models have shown that ischaemic cardiac myocytes in watershed viable zones of a myocardial infarction exhibited reperfusion-dependent expression of IL-6 mRNA after reperfusion [150, 151].

##### Procalcitonin

Procalcitonin (PCT) is a 116-amino acid peptide that has an approximate molecular weight of 14.5 kDa and belongs to the calcitonin (CT) superfamily of peptides [152]. It is a biomarker that exhibits greater specificity than other pro-inflammatory markers in identifying patients with sepsis [152].

In transplantation, PCT was used to differentiate bacterial infection from organ rejection. PCT levels are elevated in both conditions but significantly higher in the presence of infections [153, 154]. It has important prognostication value in heart transplantation with low levels signifying an uneventful course, and higher value indicating an increased mortality in the early post-operative period [155]. Venkateswaran et al. noted that elevated donor serum PCT levels were associated with poorer donor cardiac index and worse biventricular function despite early donor management during pre-retrieval optimisation [144]. The authors demonstrated that pre-optimisation baseline PCT levels of less than or equal to 2 ng mL^−1^ was a potentially useful tool in predicting the end-management heart usability for transplantation. Similar findings were noted by Wagner and colleagues with increased 30-day mortality and early graft dysfunction in hearts utilised from donors with elevated PCT levels [156]. The increased early graft failure rate was also noted in renal [157], but not in liver [158] or lung transplantation [159].

##### Neutrophil to lymphocyte ratio

Both NLR and platelet to lymphocyte ratio (PLR) have been used as markers of inflammation with prognostic value in coronary artery disease [160, 161] and other conditions. Neutrophil to lymphocyte ratios (NLR) have been shown to be independently related to mortality in patients hospitalised for acute heart failure with LVSD [162]. Implantation of LVAD in patients with heart failure showed reversibility of NLR which reflects the reversal of various HF-mediated inflammatory processes [163].

A group in Argentina studied the relationship between the two ratios and survival after heart transplantation [164]. They noted NLR (baseline and at 6 h) to be a good predictor of early mortality post-heart transplantation but not PLR. The number of patients in the single-centre study was relatively small (*n* = 111). A Polish single-centre study noted similar findings in their renal transplant cohort, with NLR showing good predictive value of early graft dysfunction [165]. Another recent study showed good correlation between NLR and survival after ECMO institution in non-transplant patients presenting with cardiogenic shock [166]. However, patients in the increased NLR ratio arm of this study were also significantly older with a higher blood urea nitrogen levels.

#### Troponin

Cardiac troponins are regulatory proteins that control the calcium-mediated interaction between actin and myosin. The measurements of serum cardiac troponin I (cTnI) and cardiac troponin T (cTnT) have shown to be sensitive and specific markers of myocardial damage [167].

The pathophysiology of brainstem death results in a catecholamine storm that is believed to cause transient myocardial ischaemia and injury [168]. Circulating cardiac troponin concentrations may therefore be elevated in the donor.

Several studies however have shown that elevated serum troponin levels in donors may predict adverse outcomes post-transplantation. Increasing cTnT levels have been shown to be associated with a reduction in left ventricular ejection fraction in the donor [169]. Potapov et al. showed in a single-centre observational study that increased donor cTnT levels were associated with an increased rate of early allograft failure [170]. They showed a similar association with PCT indicating a coexistent pro-inflammatory state in these donors. PCT and cTnT levels however showed poor specificity. There was also no correlation between PCT and cTnT values and there was no significant interaction between the two markers using a logistic regression model (*P* = 0.28). Vijay et al. conducted a similar study, using endomyocardial biopsy proven rejection at 1 year as the primary endpoint. They noted a linear correlation between grade of rejection at 1-year and donor cTnT levels [171]. Another study by Potapov et al. showed an association between increasing cTnI levels in donors to early graft dysfunction post-transplantation [172].

In donors with subarachnoid haemorrhages, cTnI was a good indicator for left ventricular dysfunction, however this was reversible, and did not affect outcomes post-transplant [173, 174]. Boccheciampe et al. noted that cTnI values in donors were not associated with PGD or post-transplant survival [175]. Other larger studies of donor serum troponin have shown no association between elevated levels and PGD [176, 177].

A recent study of cTnI in the preservation solution (University of Wisconsin solution and Custodiol) during transportation of heart grafts however showed that elevated cTnI (scaled to the corresponding LV mass) was predictive of post-transplant PGD [178]. They also noted there was poor correlation between preservation fluid cTnI levels and donor serum cTnI levels. They raised the possibility that incomplete myocardial preservation may play a role in PGD pathogenesis. However, there were only 43 patients in the study, and only ischaemic time was noted to be a predictor of PGD in their logistic regression model.

#### Brain natriuretic peptide/N-terminal pro–B-type natriuretic peptide

Brain natriuretic peptide (BNP) and its amino-terminal pro-fragment, N-terminal pro–B-type natriuretic peptide (NT-proBNP) are commonly used for diagnosis and prognostication in heart failure [179, 180]. They are released by the myocardium in response to increasing ventricular wall stress [181]. They have been shown to correlate well with ventricular dilation [181], adverse remodelling [182] and death after acute myocardial infarction [183]. Dronavalli et al. showed that elevated NT-proBNP correlate well to poor echocardiographic and haemodynamic findings of cardiac function in potential DBD donors [184]. Vorlat et al. linked increased BNP levels to a lower cardiac output post-transplant and a prolonged hospital stay [185]. They noted that a donor serum BNP of > 160 pg/mL had 89% accuracy to predict poor cardiac performance in the recipient (cardiac index < 2.2 L/min/m^2^). The authors however could not show a correlation between post-operative inotropic support and BNP levels.

#### Others

##### SWItch/sucrose non-fermentable, a matrix-associated, actin-dependent regulator of chromatin subfamily a-like 1

SWItch/sucrose non-fermentable, a matrix-associated, actin-dependent regulator of chromatin subfamily a-like 1 (SMARCAL1) is an intracellular protein that acts as a DNA-dependent ATPase involved in transcription, DNA repair and chromatin dynamics [168]. Aharinejad et al. noted that elevated serum SMARCAL1 levels in their cohort of 336 donors were predictive of recipient PGD [186]. In addition, SMARCAL1 levels correlated well with survival at 3 months, 1-year and 5-year survival. Using a donor serum cutoff of ≥ 1.25 ng/mL, they demonstrated a 96% sensitivity and 88% specificity for predicting PGD. Pre-and post-aortic donor cross-clamp serum SMARCAL1 concentrations were the best markers of PGD risk. To date, no other validation studies have been performed.

##### Donor myocardial hypoxia-inducible factor-1α

Hypoxia-inducible factor (HIF)-1 is a heterodimeric α, β transcription factor that mediates tissue responses to hypoxia [187]. Aharinejad et al. performed a series of assays using 857 donor LV myocardial biopsies obtained before and after aortic cross-clamping in the donor, and at 10, 30 and 60 min following reperfusion [188]. In the cDNA array, only HIF-1α mRNA expression after aortic cross-clamping in donors and at 10 min following the release of the aortic cross-clamp in the recipient were significant predictors of PGD. The authors hypothesise that the release of cytokines and inflammatory chemokines activated by ischaemia and reperfusion injury reach the highest peaks prior to cross-clamping and just after reperfusion. Other studies have demonstrated the protective signalling of HIF-1 against ischaemia-reperfusion injury in the heart [189, 190]. To date however, there have been no further studies linked HIF-1α to PGD.

##### Serum exosome proteomics

Several published abstracts have highlighted the role of serum exosome proteomic analysis in the recipient as a potential biomarkers for PGD [191, 192]. Giangreco et al. noted that pre-transplant serum exosome analysis revealed an inflammatory phenotype and complement activation in patients who later developed PGD which could be identified by the absence or presence of the biomarkers alone. Fine et al. showed this could be further used to differentiate patients who would later develop PGD-RV from PGD-LV. In their study, significant upregulation of angiotensinogen (AGT) and adiponectin (ADIPOQ) signalling pathways were noted in the PGD-LV group whereas hepatic growth factor activator (HGFAC) and insulin-like growth factor-binding protein 3 (IGFBP3) pathways were both upregulated in the PGD-RV group. The significance of these findings may hopefully shed light into the pathogenesis of PGD in the near future.

### Prevention of PGD

A single administration of cold flush preservation fluid remains the gold standard for myocardial protection during transplantation. It confers reliable protection for a limited amount of ischaemic time in young donor hearts [6]. The increasing use of extended criteria donors however necessitate more aggressive protection strategies to attenuate the ischaemic effects on hearts. In a study conducted by the Hamburg group, they noted that the use of a leukocyte depleting filter alongside additional regular antegrade administration of Buckberg cold blood cardioplegia in intervals of 20 min resulted in a reduction of PGD rates [193]. Similar findings were noted by a group from the Czech Republic, utilising continuous cold blood cardioplegia followed by controlled reperfusion of warm blood [194]. The Glasgow group utilised a similar variation with continuous antegrade perfusion in a contemporary cohort of patients, resulting in a significant reduction in PGD rates compared to a historical group of patients and the national UK cohort [195]. The exact mechanisms by which additional cardioplegia administration reduces PGD remain uncertain. It may play a role in pre-conditioning, mitigating the impact of reperfusion or preventing generation and propagation of inflammatory pathways [196].

### Treatment and management of PGD

Treatment of PGD thus far is still primarily supportive care. In a consensus statement by Kobashigawa et al., the treatment and management of PGD across five high volume transplant centres were evaluated. PGD is initially managed by using inotropic support using catecholamines and phosphodiesterase inhibitors. The most common escalation therapy follow inotropic use is using an intra-aortic balloon pump. Following this, advanced mechanical support is initiated which is usually directed by the expertise of the transplant units themselves. The most common mode of support is extracorporeal membranous oxygenation with both central and peripheral cannulation strategies utilised. The heart should be allowed to eject to prevent stasis and thromboembolic complications.

For primary RV-PGD, nitric oxide may be used to reduce the pulmonary vascular resistance. Following a period of support on ECMO, a short-term ventricular assist device is then implanted depending on the level of support needed (LVAD, RVAD, BiVAD ± oxygenator, total artificial heart). Where possible, the patient is then re-evaluated for a redo-orthotopic heart transplantation.

#### Levosimendan

Levosimendan is a calcium sensitising agent and inodilator which increases cardiac contractility. Its primary mode of action is by increasing the sensitivity of troponin-C to calcium during systole thereby increasing cardiac performance without increasing myocardial oxygen consumption. It also reduces peripheral vascular resistances by opening of adenosine triphosphate–dependent K+ channels [197]. It was initially used for treatment of cardiogenic shock post-acute myocardial infarction [197]. However, SURVIVE, a multicentre RCT evaluating levosimendan vs dobutamine in acute decompensated heart failure, revealed no differences in survival outcomes between the two groups [198].There was an initial reduction in plasma B-type natriuretic peptide level in patients in the levosimendan group compared with patients in the dobutamine group. Florian Weis and colleagues report a case series of 12 patients with PGD who received 0.1 μg/kg/min levosimendan for PGD with a centre-specific definition of LVEF < 30% on transesophageal echocardiogram with a combination of adrenaline > 0.1 μg/kg/min and the milrinone > 0.3 μg/kg/min [199]. Eleven of the 12 patients survived beyond 30 days, with significant reductions in inotropic support without requiring mechanical circulatory support. However, follow-up studies by the same group showed significantly lower 1-year and 3-year survival rates [200].

The inotropic effects of levosimendan may often take hours to develop, which may be offset by its potent vasodilation capacity [201]. It may however also play a role in protecting cardiomyocytes against ischaemic/reperfusion injury by activating adenosine triphosphate-sensitive potassium channels in mitochondria as shown by numerous studies although to date, no studies have been done on pre-or post-conditioning in a heart transplant setting [202–206].

#### Plasmapheresis

Plasmapheresis is a process by which whole blood is passed through a filter to separate the plasma components from the larger cellular components of red blood cells, white blood cells and platelets [207]. Its use in transplantation was primarily for hyperacute or acute humoral rejection, whereby adjunctive therapeutic plasma exchange has been used alongside immunosuppression and intravenous immunoglobulins to improve survival of incompatible organs compared to compatible organs [208]. Plasmapheresis rapidly reduces the circulating antibodies and thereby improves cardiac function [209]. Plasmapheresis has been used for primary allograft dysfunction after liver transplantation with encouraging results [210, 211]. A study by a group in Taiwan revealed treatment with pulsed steroid and plasmapheresis improved the ejection fraction and NYHA status of > 70% of the patients treated [212]. However, the definitions of PGD in this cohort were not discernible from acute rejection due to the study methodology and lack of biopsy-proven rejection at the time of treatment as treatment was started empirically in all 35 patients who were in NYHA functional class III or IV requiring pharmacological or mechanical circulatory support. The Cedars-Sinai Heart Institute group performed a similar study in patients with severe PGD. In their cohort of 15 patients, 66% of whom underwent plasmapheresis post-transplantation had significantly improved outcomes compared to the 33% of severe PGD patients who did not. They concluded that the potential role of inflammatory molecule depletion in these patients may play a role in the pathophysiology of PGD [213]. The exact role of plasmapheresis in PGD is still speculative and may potentially be clearer with ongoing research.

### Conclusion

PGD is the leading cause of early morbidity and mortality following heart transplantation. It is thought to be multifactorial in origin and several risk factors implicated. The search for an accurate biomarker however remains elusive. Treatment options to date remain supportive with no definitive pharmacological agents identified as of yet. Plasmapheresis may have a role in depletion of inflammatory chemokines and cytokines for treatment of PGD.
